# Synthesis of F127-GA@ZnO nanogel as a cisplatin drug delivery pH-sensitive system

**DOI:** 10.1039/d4ra06514j

**Published:** 2024-11-04

**Authors:** Nguyen Ngoc Son, Vu Minh Thanh, Nguyen Thi Huong

**Affiliations:** a Institute of Chemistry and Materials 17 Hoang Sam, Cau Giay Hanoi Vietnam nguyenhuong0916@gmail.com

## Abstract

In this study, a novel drug delivery system based on zinc oxide nanoparticles (ZnO NPs) was developed for the enhanced delivery of cisplatin (CPT) to improve cancer treatment. The ZnO NPs were synthesized from guava leaf extract and then surface-functionalized with gallic acid (GA) to improve their biocompatibility and drug loading capacity. Pluronic F127, a biocompatible polymer, was then conjugated to the GA-modified ZnO NPs to further enhance their stability and cellular uptake. The resulting NPs were characterized by various techniques, including FT-IR, UV-Vis, SEM, TEM, ^1^H NMR, and DLS. The drug loading and release profiles of CPT from the NPs were investigated, showing high CPT loading capacity and pH-dependent release behavior. The *in vitro* cytotoxicity of the NPs was evaluated against various cancer cell lines, demonstrating enhanced cytotoxicity compared to free CPT. Overall, this study highlights the potential of GA and Pluronic-modified ZnO NPs as a promising drug delivery system for enhanced CPT delivery and improved cancer therapy.

## Abstract 2

In this study, a novel drug delivery system based on zinc oxide nanoparticles (ZnO NPs) was developed for the enhanced delivery of cisplatin (CPT) to improve cancer treatment.

## Graphical abstract

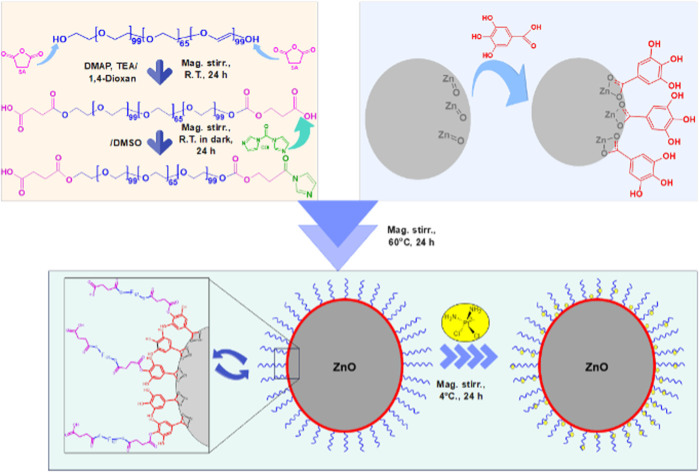

## 1. Introduction

Cancer, a disease characterized by uncontrolled cell growth and the ability to invade surrounding tissues, continues to be one of the most significant public health challenges globally.^1^ Despite considerable advancements in cancer diagnosis and treatment, the incidence and mortality rates remain alarmingly high, highlighting the urgent need to develop more effective and safe therapies.^2,3^ Cisplatin (CPT), a platinum complex widely used as a chemotherapeutic agent for cancer treatment, has demonstrated efficacy against a variety of cancers, including lung, ovarian, testicular, and bladder cancers.^4^ However, the clinical use of cisplatin is significantly hampered by several inherent limitations, such as severe systemic toxicity, poor water solubility, and lack of selectivity towards cancer cells. These limitations often lead to adverse side effects, including nephrotoxicity, neurotoxicity, and myelosuppression, which can severely impact the patient's quality of life and limit the overall therapeutic efficacy.^5–7^ Furthermore, drug resistance is also a major issue in CPT-based cancer therapy, reducing treatment efficacy and increasing the risk of relapse.^8–11^

To address these limitations and improve the therapeutic efficacy of cisplatin, researchers have actively explored the use of nano-drug delivery systems, which are capable of encapsulating and delivering drugs to tumor sites in a controlled manner, thereby minimizing side effects and enhancing therapeutic efficacy.^12,13^ To date, a diverse array of nanocarrier platforms has been explored for the delivery of CPT, encompassing liposomes,^14–18^ polymeric nanoparticles (nanogels, micelles, and dendrimers)^15–18^ and inorganic nanoparticles (typically metal or metal oxide-based).^19–27^ Each of these drug delivery systems presents a unique set of advantages and drawbacks. Inorganic nanoparticles, notably, are frequently distinguished by their inherent stability and amenability to surface functionalization, facilitating the fabrication of sophisticated architectures such as core–shell nanoparticles or mesoporous nanoparticles.^28,29^ Among various inorganic nanoparticles, zinc oxide nanoparticles (ZnO NPs) have emerged as a promising drug carrier material due to their biocompatibility, biodegradability, and inherent antibacterial activity.^30–32^ ZnO NPs have been shown to exhibit selective toxicity towards cancer cells through multiple mechanisms, including the generation of reactive oxygen species (ROS), release of zinc ions, and induction of apoptosis.^33,34^ Furthermore, ZnO NPs can be synthesized using various green methods, such as using plant extracts, providing a sustainable and eco-friendly approach for nanoparticle production.^26,35,36^ Zinc oxide nanoparticles (ZnO NPs) when combined with medicinal agents have been shown to greatly improve the therapeutic effectiveness of these medications. C. Hu and W. Du^37^ investigated the combination of ZnO NPs with cisplatin (CPT) and gemcitabine (Gem) for the treatment of non-small cell lung cancer (NSCLC). Their findings demonstrated that this combination significantly promoted apoptosis and reduced the viability of A549 cells compared to controls (individual drugs CPT or Gem, or individual drug combinations ZnO(CPT), ZnO(Gem), or the combination of drugs (CPT/Gem) without ZnO NPs). This study suggests that ZnO NPs can potentiate the therapeutic efficacy of chemotherapeutic agents, highlighting their promising potential in tumor treatment. S. Alipour *et al.*^38^ conducted a comparative study on the apoptosis-inducing efficacy of ZnO NPs, alantolactone (ALT), and cisplatin (CPT), as well as their binary and ternary combinations, against the SKOV3 ovarian cancer cell line. A series of MTT assays, real-time PCR analyses, and intracellular ROS level assessments were performed. The results revealed that the ternary combination of ZnO NPs, CPT, and ALT markedly enhanced the therapeutic effect compared to the individual agents or their binary combinations. This further underscore the potential of utilizing ZnO NPs in combination therapies for the treatment of SKOV3 ovarian cancer cells. The results regarding the combination therapy of ZnO NPs have yielded positive outcomes. However, to design a complete formulation, the drug loading capacity of ZnO NPs needs to be improved. Y. Guo and Z. Sun^39^ fabricated a targeted drug delivery system to co-deliver CPT and docetaxel (DOC) based on surface-modified ZnO NPs, aiming to enhance therapeutic efficacy against nasopharyngeal carcinoma cells. Accordingly, PEG-coated ZnO NPs with a particle size of approximately 43 nm were synthesized and subsequently conjugated with folic acid (FA) molecules. The resulting FA-PEG-ZnO material was then loaded with CPT and DOC. The obtained drug loading efficiencies for CPT, DOC, and the dual CPT/DOC system were 41.5%, 43.45%, and 47.14%, respectively. A marked improvement in cellular uptake of CPT and DOC was observed. Recently, S. Y. Mohamed *et al.*^40^ synthesized ZnO NPs and obtained nanocomposite materials by combining them with a range of agents, including mandelic acid (ZnO-M), graphene oxide (ZnO-GO), and chitosan (CS-ZnO). However, the drug loading efficiency of these materials appears to be less than desirable, with values of 37.8% (ZnO/CPT), 33.56% (ZnO-M/CPT), 17.76% (ZnO-GO/CPT), and 22.7% (CS-ZnO/CPT), respectively. Not only did the drug loading efficiency decrease, but the toxicity of the drug delivery system to MCF-7 cancer cells also declined, with observed IC50 values of 2.12 μg mL^−1^ (ZnO/CPT), 6.59 μg mL^−1^ (ZnO-M/CPT), 15.1 μg mL^−1^ (ZnO-GO/CPT), and 10.7 μg mL^−1^ (CS-ZnO/CPT). Thus, the surface modification of ZnO NPs in this study did not yield positive results. This suggests that the choice of agent used to structure the surface of ZnO NPs plays a crucial role.

Gallic acid (GA) is a polyphenol compound abundantly found in various natural fruits and vegetables such as chestnuts, gallnuts, clove leaves, and tea leaves.^41,42^ GA is a potent antioxidant, capable of neutralizing harmful free radicals and exhibiting a wide range of biological activities. Consequently, it has been extensively researched for applications in the biomedical field.^43,44^ GA and its derivatives have been suggested to induce cytotoxicity in cancer cells while remaining safe for healthy cells.^45,46^ They are also believed to cause cancer cell death through various pathways.^47–49^ With a chemical structure comprising a carboxylic group and three hydroxyl groups directly attached to an aromatic ring, their chemical activity is quite diverse. GA is favoured for surface functionalization of various inorganic nanoparticles such as Au,^41,42^ Fe_3_O_4_,^50,51^ Al_2_O_3_,^52^ Ag,^53^ ZnO.^54^

Poloxamers, a family of triblock copolymers consisting of a central hydrophobic polypropylene oxide (PPO) block flanked by two hydrophilic polyethylene oxide (PEO) blocks, have been widely used as nanogel-forming materials due to their excellent biocompatibility, micelle-forming ability, and thermo-responsive properties.^55^ Specifically, poloxamer 407 (Pluronic® F127), a U.S. Food and Drug Administration (FDA)-approved poloxamer, has demonstrated reversible gelation properties at body temperature, making it an attractive candidate for drug delivery applications.^56^ F127 can self-assemble into micelles at concentrations above the critical micelle concentration (CMC), forming a hydrophobic core for encapsulating hydrophobic drugs and a hydrophilic shell for enhancing colloidal stability and prolonging blood circulation.^57^ Moreover, F127 has been shown to exhibit enhanced permeability and retention (EPR) effect, a phenomenon in which nanoparticles tend to accumulate at tumor sites due to increased vascular permeability and impaired lymphatic drainage. This feature enables targeted drug delivery to tumor cells, further enhancing therapeutic efficacy and minimizing off-target toxicity.^58,59^

In this study, we developed a novel pH-sensitive cisplatin drug delivery system based on F127-GA@ZnO nanogel. First, ZnO nanoparticles synthesized by an environmentally friendly green method in our previous study^60^ were used, followed by surface modification with GA to improve dispersion and conjugation. Then, F127 tails were grafted onto the GA@ZnO surface *via* chemical bonding to form F127-GA@ZnO nanogel. The nanogels were thoroughly characterized for their physicochemical properties and evaluated for cisplatin loading capacity, pH-sensitive drug release, and toxicity against various cancer cell lines.

This study aimed to develop an innovative and effective cisplatin drug delivery system based on F127-GA@ZnO nanogels, combining the advantages of both polymers and inorganic nanoparticles. We anticipate that this novel drug delivery system will exhibit high drug loading capacity, controlled drug release at tumor sites, and enhanced anticancer activity compared to free cisplatin, ultimately leading to improved therapeutic outcomes and reduced side effects for cancer patients.

## 2. Experimental

### 2.1. Material

Pluronic F127 (F127, MW 12,600 Da), BioReagent, 4-dimethylaminopyridine (DMAP, ReagentsPlus, ≥99%), and 1,1′-carbonyldiimidazole (CDI, ≥97.0%), cisplatin (CPT, pharmaceutical primary standard) were purchased from Sigma Aldrich (USA). Solvents including absolute ethanol (EtOH, ≥99.8%), dimethyl sulfoxide (DMSO, ≥99.9%), triethylamine (TEA, ≥99.5%), 1,4-dioxane (≥99.5%), chloroform (≥99.5%), and diethyl ether (≥99.5%) were analytical grade and supplied by Fisher BioReagents™. Succinic anhydride (SA, Reagents) and gallic acid (GA, ≥99%) were obtained from Biobasic (Canada). All chemicals were used as received without further purification. ZnO NPs were synthesized in our laboratory from zinc acetate dihydrate (Emsure ACS, Merck – Germany) and guava leaf extract following a previously published procedure.^60^ The as-prepared ZnO NPs were characterized by X-ray diffraction (XRD) and found to be approximately 30–40 nm in size, quasi-spherical in shape, and possessing a hexagonal wurtzite crystal structure.

### 2.2. Synthesis and characterization

#### 2.2.1. Functionalization of ZnO with gallic acid (GA@ZnO)

The amount of 0.170 g (1 mmol) of GA was completely dissolved in 150 mL of double-distilled water (DW) and added 0.81 g (10 mmol) of ZnO NPs. The reaction mixture was sonicated for 90 minutes. The solid product was then collected by centrifugation (10 000 rpm for 5 minutes) and washed several times with DW to remove unreacted GA. The fine powder of GA-functionalized ZnO (GA@ZnO) was then obtained by vacuum drying at room temperature for 24 h.

#### 2.2.2. Carboxylation of F127

The terminal hydroxyl group of F127 was converted to a carboxyl group following the procedure of J. L. Tian *et al.*^61^ Briefly, 9.45 g (0.75 mmol) of F127, 0.56 g (3 mmol) of SA, and 0.5 g of DMAP were dissolved in 30 mL of 1,4-dioxane. Subsequently, 0.5 mL of TEA was added. The reaction was carried out at room temperature for 24 h under magnetic stirring. After the reaction, the 1,4-dioxane solvent was removed under reduced pressure. The remaining solid was redissolved in chloroform and precipitated in cold diethyl ether to remove unreacted components. The solid was collected by vacuum filtration, and residual chloroform was removed by vacuum drying at room temperature. The successful carboxylation of F127 (F127-COOH) was confirmed by ^1^H-NMR and FT-IR spectroscopy.

#### 2.2.3. Synthesis of F127-GA@ZnO

The grafting of F127-COOH to GA@ZnO was carried out by the ester linkage reaction between the carboxyl group and the hydroxyl group. It was carried out according to a previously published procedure^62^ with some modifications. Specifically, 2 g of F127-COOH was completely dissolved in 50 mL of DMSO, 0.1 g of CDI was added and stirred for 24 h in the dark at room temperature to activate the carboxyl group. Then, 0.5 g of GA@ZnO was added, heated to 60 °C and stirred for 24 h. The reaction mixture was centrifuged (10 000 rpm, 5 min), and the resulting solid was washed several times with EtOH to remove the unreacted residue. F127-GA@ZnO was obtained by vacuum drying the solid product for 12 h at room temperature.

#### 2.2.4. Characterization of materials

Fourier-transform infrared (FT-IR) spectra were recorded using the KBr pellet technique on a Bruker Tensor II spectrometer, with a scanning range of 400–4000 cm^−1^ and a resolution of 4 cm^−1^. Proton nuclear magnetic resonance (^1^H-NMR) spectra were acquired on a Bruker AvanceNEO 600 MHz spectrometer, employing appropriate solvents as indicated for each measurement. Ultraviolet-visible (UV-Vis) spectra were obtained using a Genesys 10S spectrophotometer. Both scanning electron microscopy (SEM) images and energy-dispersive X-ray spectroscopy (EDS) spectra were recorded on a JEOL-JMS 6490 instrument. Transmission electron microscopy (TEM) images were captured on a JEM 2100 microscope. The hydrodynamic diameter and zeta potential of the materials were measured using a NanoPlus HD analyzer. Thermogravimetric analysis (TGA) coupled with derivative thermogravimetry (DTG) was performed on a Labsys Evo instrument under an air atmosphere with a heating rate of 10 °C min^−1^.

### 2.3. Evaluation of cisplatin (CPT) loading and release capacity

To prepare the drug-loaded nanocarrier, 250 mg of F127-GA@ZnO was dispersed in 250 mL of DW using sonication for 15 minutes (solution A). Concurrently, a predetermined mass of cisplatin (CPT) was dissolved in 100 mL of DW under light-protected conditions and continuous stirring (solution B). The CPT loading process involved the slow, dropwise addition of solution B into solution A at a rate of 50 mL hour^−1^, maintained under magnetic stirring at 4 °C for 24 h. Subsequently, the mixture was transferred to a dialysis membrane with a molecular weight cut-off of 3500 Da and dialyzed against DW at room temperature for 8 h, with the dialysis medium replaced every 2 h. The final product, F127-GA@ZnO/CPT, was obtained after lyophilization. The CPT loading capacity of F127-GA@ZnO was then assessed by calculating the entrapment efficiency (EE) and drug loading (DL) using the following formulas:1
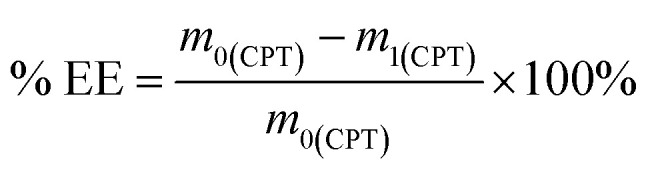
2
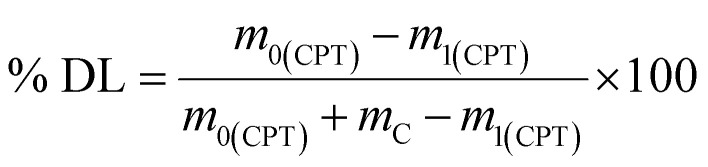
where: % EE: entrapment efficiency of CPT (%), % DL: drug loading of CPT (%), *m*_0(CPT)_: initial mass of CPT (mg), *m*_1(CPT)_: mass of free CPT (mg), *m*_C_: mass of F127-GA@ZnO carrier (mg).

### 2.4. Evaluation of CPT release characteristics of F127-GA@ZnO

To investigate the drug release kinetics, 20 mg of F127-GA@ZnO/CPT was dispersed in 20 mL of PBS buffer solution within a 50 mL glass beaker, aided by magnetic stirring to ensure homogeneity. The entire suspension was then transferred to a dialysis bag with a molecular weight cut-off of 3500 Da. Dialysis was performed against 100 mL of PBS buffer at the same pH, maintained at 37 °C. Aliquots of 3 mL were withdrawn at predetermined time intervals (1, 3, 6, 9, 12, 24, 36, 48, and 72 h), with the volume replenished each time using an equal amount of the corresponding buffer solution. The drug release capacity of the material was evaluated at pH values of 7.4, 6.4, and 5.5. The amount of drug released was quantified using the following formula:3
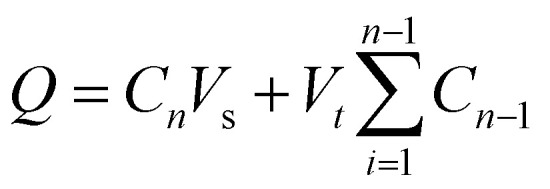
where: *Q* is the amount of CPT released from the nanocarrier (mg). *C*_*n*_ is the concentration at time *t* (mg mL^−1^); *V*_s_ is the volume of PBS (mL); *V*_*t*_ is the volume of the sample (mL); 
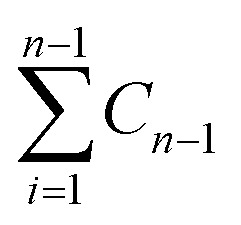
 is the concentration of CPT released over time (mg mL^−1^).

The concentration of CPT in solution was determined by UV-Vis spectroscopy at 706 nm, utilizing a standard curve generated from CPT solutions complexed with *o*-phenylenediamine (OPDA).^63^ The drug release kinetics were subsequently analyzed employing the Korsmeyer–Peppas model (eqn (4)) and the Higuchi model (eqn (5)).^64,65^4
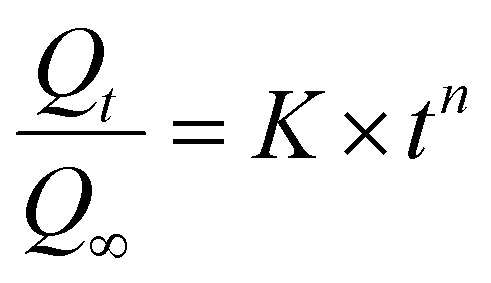

5 *Q*_*t*_ = *K*_H_ × *t*^1/2^

Here, *Q*_*t*_ is the amount of drug released at time *t*, and *Q*_∞_ is the total amount of drug released at the end. *n*, *K*, and *K*_H_ are the diffusional exponent and the Korsmeyer–Peppas and Higuchi rate constants, respectively.

### 2.5. Cytotoxicity assessment

Cell viability was assessed using the AlamarBlue assay. Cells were seeded in DMEM (10% FBS, 1% penicillin–streptomycin) at a density of 3 × 10^4^ cells per mL in 96-well plates and incubated for 24 h at 37 °C in a 5% CO_2_ atmosphere. Subsequently, the culture medium was replaced with fresh medium containing varying concentrations of the test samples (free CPT, F127-GA@ZnO, and F127-GA@ZnO/CPT). Following a 48 hours incubation at 37 °C in 5% CO_2_, AlamarBlue reagent was added directly to each well at a 10% volume ratio, followed by gentle mixing. The plates were then incubated for an additional 1–4 h at 37 °C in 5% CO_2_. The post-incubation solution was transferred to a new 96-well plate, and fluorescence was measured at an excitation/emission wavelength of 560/590 nm. Absorbance was also read at 570 nm with a reference wavelength of 600 nm (signal remains stable for 7 h). A standard curve correlating fluorescence/absorbance units to sample concentration was constructed for quantitative analysis. Cell viability was calculated using the following formula:6
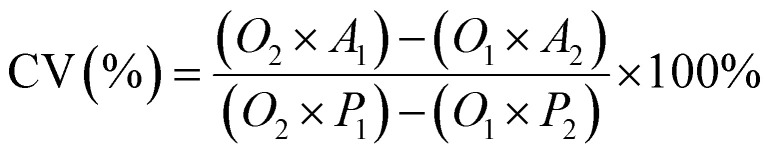
where, CV is cells viability; *O*_1_ is the molar extinction coefficient (*E*) of oxidized AlamarBlue (blue) at 570 nm; *O*_2_ is the *E* of oxidized AlamarBlue at 600 nm; *A*_1_ and *A*_2_ are the absorbance values of the experimental wells at 570 nm and 600 nm, respectively. *P*_1_ and *P*_2_ are the absorbance values of the control wells (cells and AlamarBlue without test substance) at 570 nm and 600 nm, respectively.

## 3. Results and discussion

### 3.1. Functionalization of ZnO NPs with GA

**Fig. 1:**
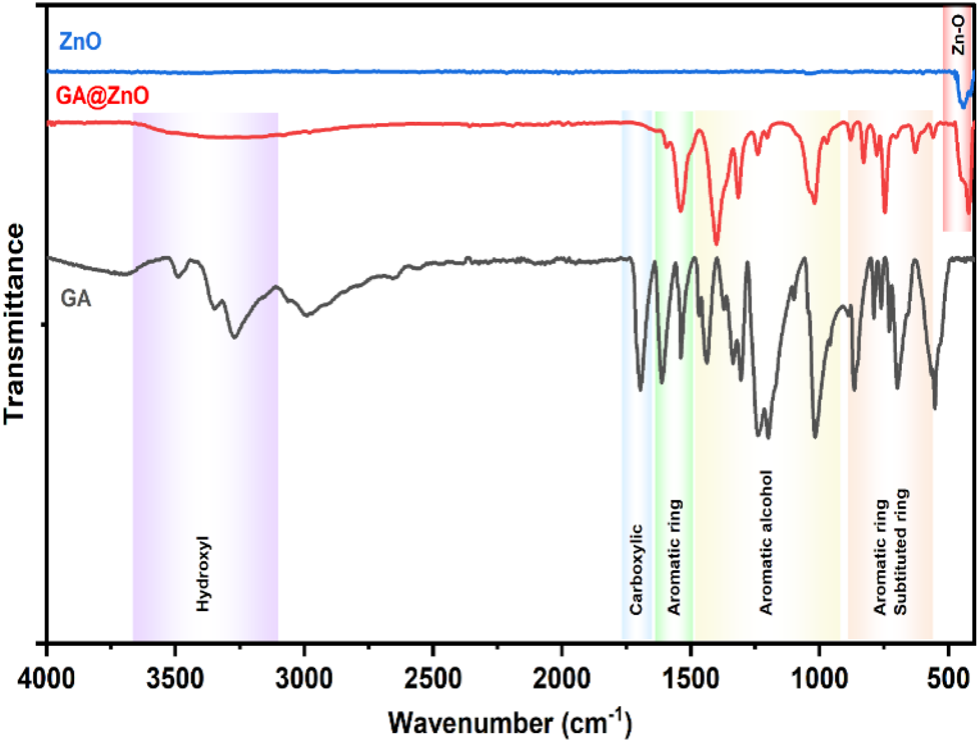
The FT-IR spectra of ZnO, GA@ZnO.

**Scheme 1:**
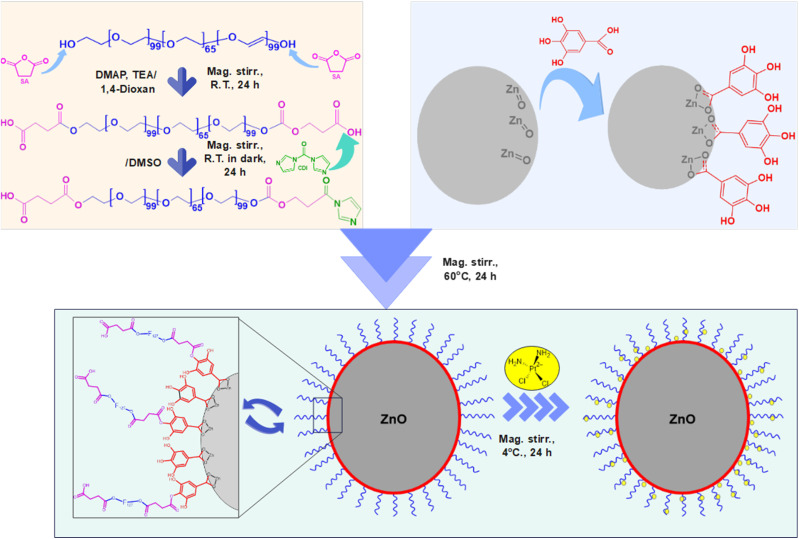
The synthesis and CPT loading (CPT) of F127-GA@ZnO nanogel.

The successful grafting of GA onto the ZnO NP surface is corroborated by the infrared spectra illustrated in Fig. 1. The FT-IR spectrum of GA displays characteristic peaks associated with hydroxyl groups (O–H bonds) linked to the aromatic ring at 3488.74 cm^−1^ and 3348.96 cm^−1^, while the O–H bond within the carboxyl group is evident at 3270.51 cm^−1^. Peaks at 1199.52 cm^−1^ and 1239.46 cm^−1^ correspond to the O–H and C–O vibrations of the aromatic alcohol moiety in GA. Notably, a prominent peak at 1695.87 cm^−1^ is ascribed to the C=O bond of aromatic carboxylic compounds. Further, vibrations of the O–H and C–O bonds in the carboxylic group are discernible at 1437 cm^−1^ and 1305.07 cm^−1^. Peaks indicative of the aromatic ring structure of GA are observed at 1613.15 cm^−1^, 1538.98 cm^−1^, 865.77 cm^−1^, and 553.41 cm^−1^.^66,67^ In the FT-IR spectrum of ZnO NPs synthesized from guava leaf extract, a sole band is observed in the 440–460 cm^−1^ region, with two characteristic peaks at 442.15 cm^−1^ and 454.99 cm^−1^, representing the Zn–O bond. The spectrum of GA@ZnO reveals the presence of almost all the characteristic bands of both ZnO and GA, albeit with some noteworthy alterations. A redshift of approximately 20 cm^−1^ is observed in the characteristic band of the Zn–O bond, potentially attributable to the influence of the GA coating on the nanoparticle surface, particularly the carboxyl group inducing changes in the Zn–O bond. Regions characteristic of the GA ring structure and its aromatic alcohol component are retained, while the carboxylic region undergoes significant modification. Most strikingly, the peak at 1695.87 cm^−1^, characteristic of the C=O bond in the carboxylic group, virtually disappears.^68^ This observation can be explained by the attachment of the carboxylic group to the ZnO NP surface, with both of its oxygen atoms participating in bond formation, thereby anchoring it to the nanoparticle surface. This finding supports the hypothesized bond formation between GA and the ZnO NP surface as depicted in Scheme 1.

**Fig. 2:**
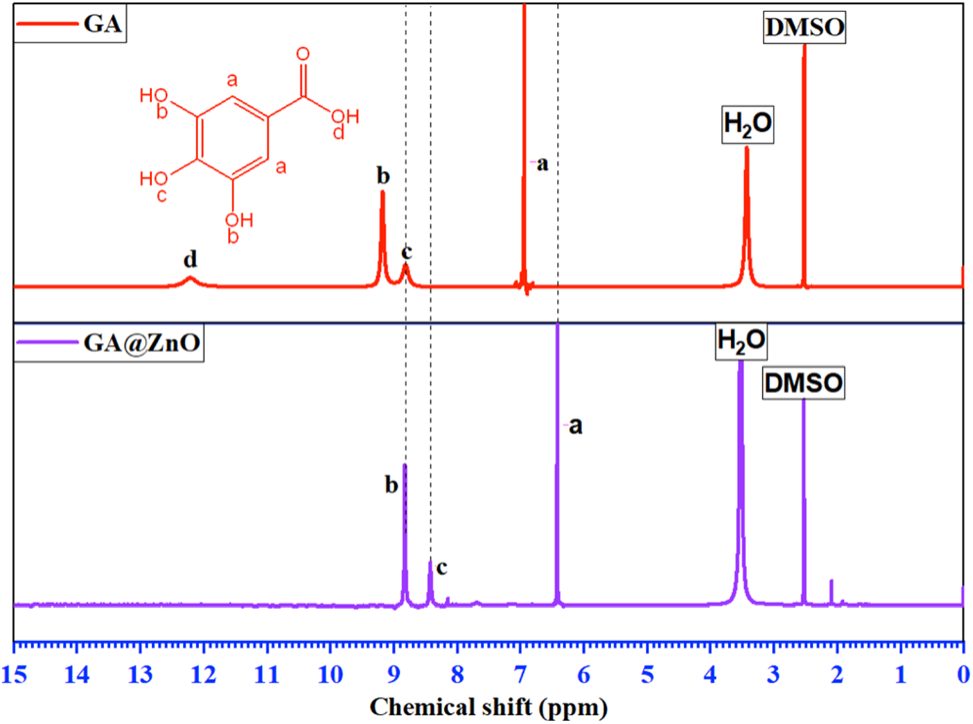
The ^1^H-NMR spectra of GA and GA@ZnO.

The ^1^H-NMR spectrum (Fig. 2) further substantiates the successful grafting of GA onto the ZnO surface *via* carboxyl group. The spectrum of GA exhibits characteristic resonance peaks at *δ*_(a)_ = 6.94 ppm, attributed to the aromatic protons (a), *δ*_(b)_ = 9.17 ppm, corresponding to the protons (b) of the –OH groups at the *meta* positions, and *δ*_(c)_ = 8.81 ppm, indicative of the protons (c) of the –OH group at the *para* position. Additionally, a distinct resonance peak at *δ*_(d)_ = 12.21 ppm is observed, representing the proton (d) within the carboxyl group.

The ^1^H-NMR spectrum of GA after its attachment to the ZnO nanoparticle surface exhibits significant alterations. Firstly, the complete disappearance of the carboxyl proton (d) signal is observed, accompanied by an upfield shift of the aromatic protons: *δ*_(a)_ = 6.41 ppm, *δ*_(b)_ = 8.82 ppm, and *δ*_(c)_ = 8.41 ppm. This observation is indicative of the anticipated shielding effect. Upon the replacement of the H^+^ proton with the Zn^2+^ cation, the weaker electron-withdrawing capacity of Zn^2+^ compared to H^+^ results in an increase in electron density on the carboxyl group. This increased electron density is delocalized into the aromatic ring, leading to the observed shielding effect. Furthermore, the magnitude of the chemical shift changes reveals a more pronounced shielding effect at the *ortho* and *para* positions, consistent with typical aromatic ring effects. In conclusion, the ^1^H-NMR results unequivocally confirm the successful grafting of GA onto the ZnO nanoparticle surface *via* its carboxyl group.

**Fig. 3:**
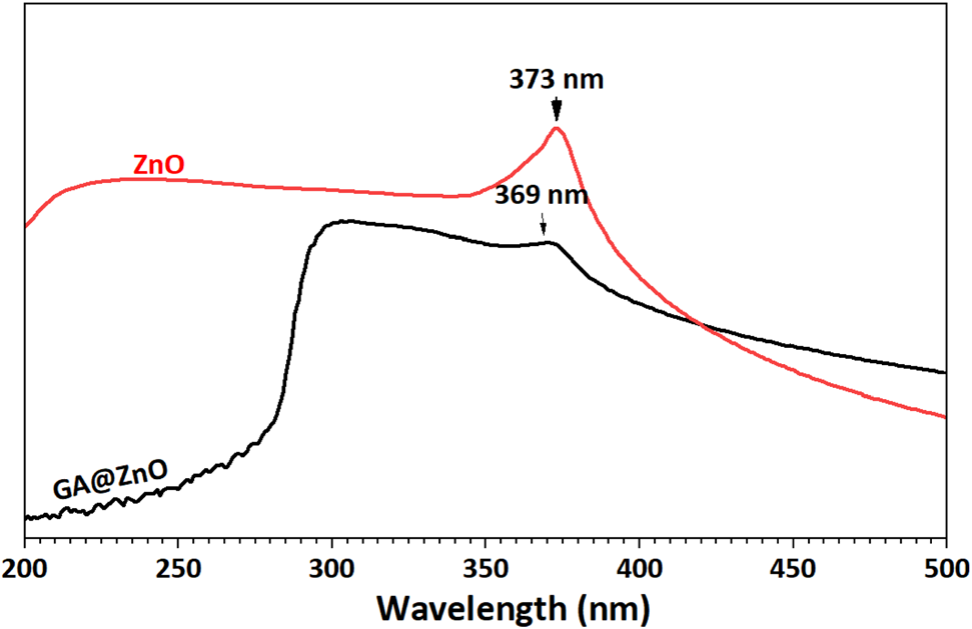
UV-Vis spectra of ZnO (black) and GA@ZnO (red).

The UV-Vis spectra of ZnO before and after modification were also recorded (Fig. 3). This pectrum reveals an absorption peak at 373 nm, characteristic of ZnO.^69,70^ Meanwhile, the UV-Vis spectrum of GA@ZnO shows a decrease in the intensity of the characteristic absorption peak, which can be attributed to the shielding effect of the organic layer on the nanoparticle surface. Additionally, a blue shift is observed in the characteristic absorption peak of ZnO, as its maximum absorption peak shifts to 369 nm. This blue shift in the UV-Vis spectrum can be attributed to the presence of electron-withdrawing substituents, which reduce the electron density in the Zn–O bond, increasing the energy gap and causing a hypsochromic shift (blue shift).^71–73^ This hypothesis is consistent in this case as the gallic cation is a strong electron-withdrawing group.

**Fig. 4:**
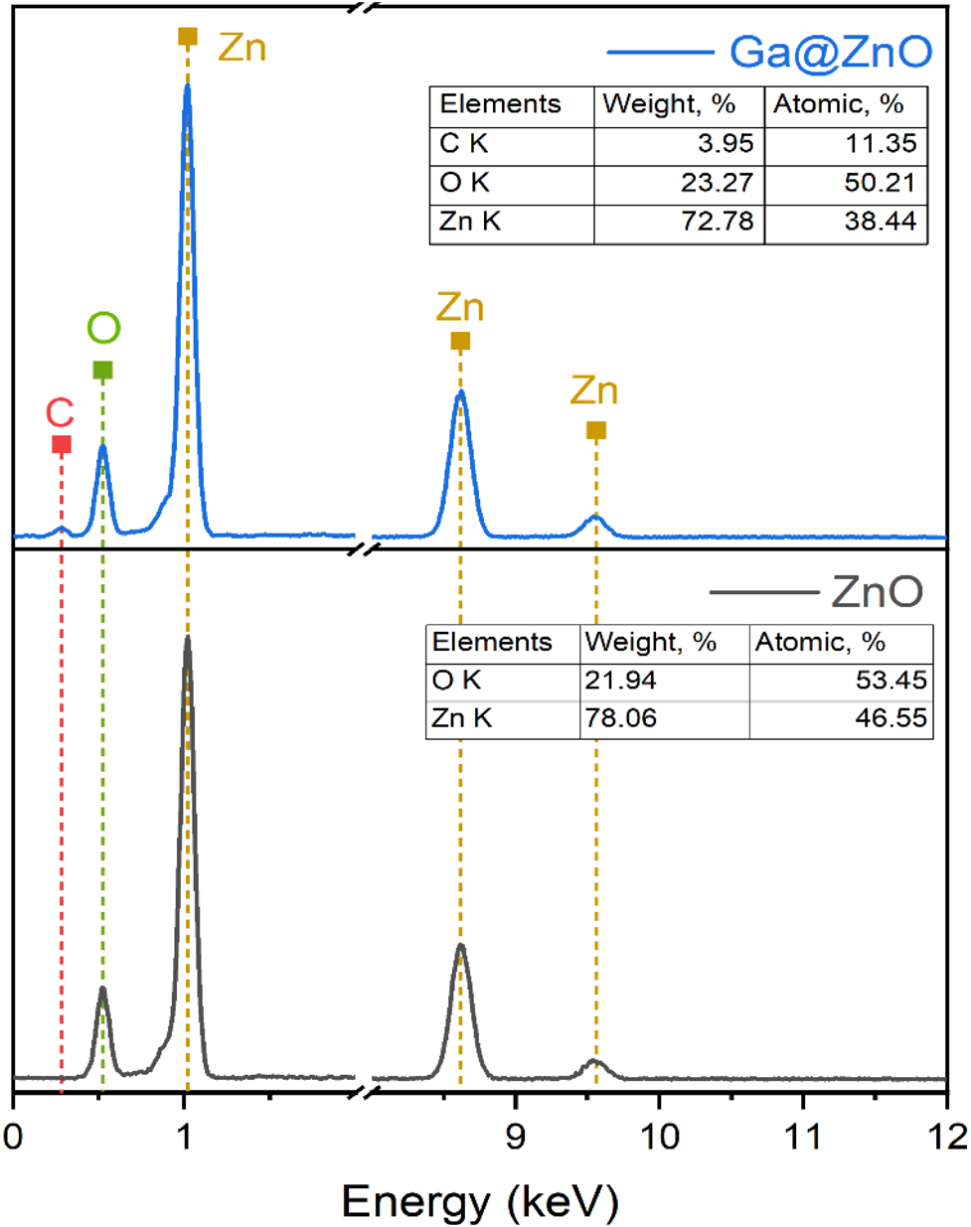
EDS spectra of ZnO NPs (bottom) and GA@ZnO (top).

The elemental composition of bare ZnO and after functionalization with GA was also determined by EDX spectroscopy (Fig. 4). The results revealed a significant presence of carbon with an atomic percentage of up to 11.35% after functionalization. Additionally, the O/Zn ratio also increased considerably in GA@ZnO compared to the uncoated ZnO.

**Fig. 5:**
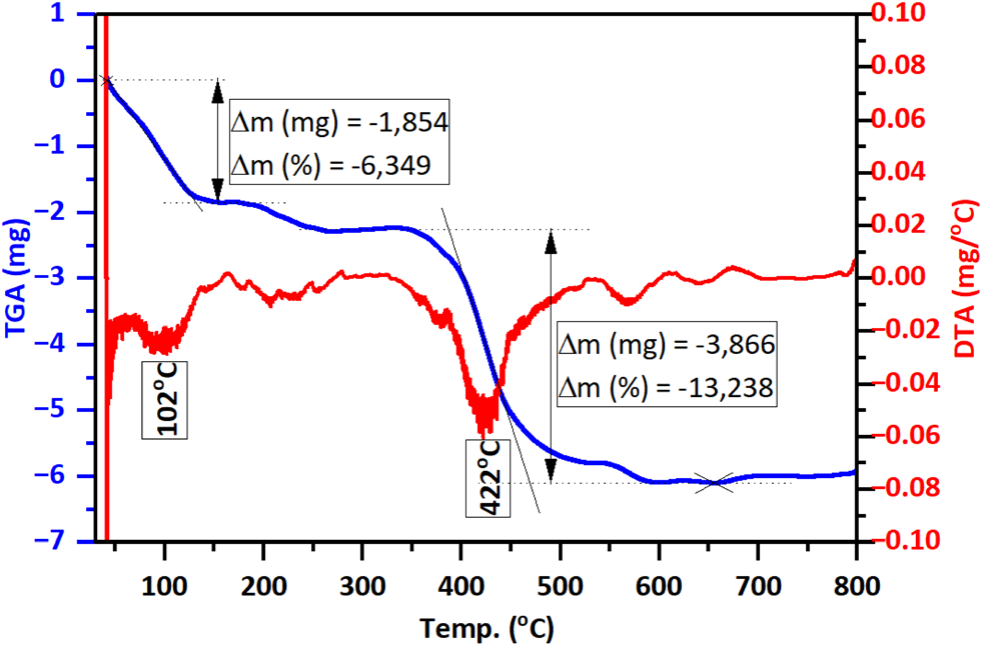
TGA/DTG thermograms of GA@ZnO.

The TGA/DTG analysis results (Fig. 5) reveal that GA@ZnO exhibits two prominent weight loss stages with increasing temperature: around 102 °C and 422 °C. The weight loss at the lower temperature region of 102 °C, amounting to 6.349%, can be attributed to the evaporation of physically/chemically adsorbed moisture on the material. Meanwhile, the significant weight loss at 422 °C, reaching 13.238%, can be assigned to the decomposition of GA grafted onto the surface of ZnO nanoparticles.

**Fig. 6:**
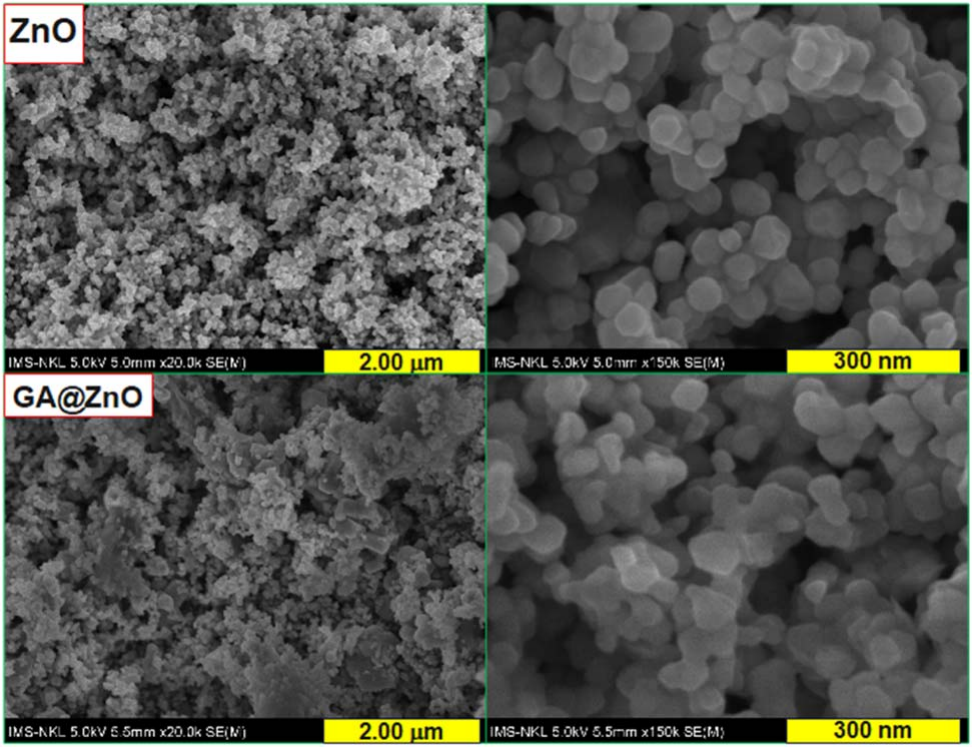
SEM images of ZnO (top) and GA@ZnO (bottom).

Examination of the morphology of the nanoparticles before and after surface modification using scanning electron microscopy (SEM) also revealed changes in their morphology. The SEM images are presented in Fig. 6. For ZnO, the nanoparticles exhibit a spherical shape with high uniformity, and the particle size is around 30–40 nm. In contrast, the nanoparticles after modification with GA show a slight change in shape, becoming less defined and spherical compared to the unmodified nanoparticles. However, the size distribution of the particles remains uniform.

### 3.2. Terminal carboxylation of F127

**Fig. 7:**
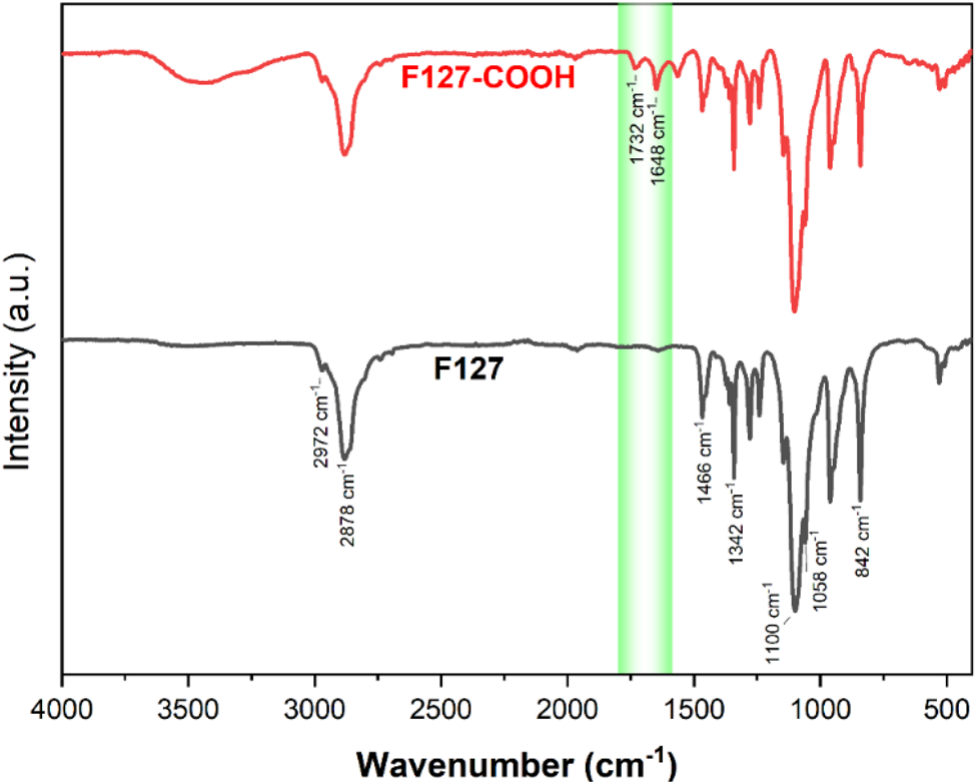
The FT-IR spectra of F127 and F127-COOH.

The successful transformation of F127's terminal hydroxyl groups into carboxylic acid functionalities *via* reaction with succinic acid (SA) was corroborated by both FT-IR and ^1^H NMR spectroscopic analyses. The FT-IR spectrum of pristine F127 (Fig. 7) displays characteristic absorption bands at 1058 cm^−1^, indicative of the C–O stretching vibration of primary alcohols, and at 842 cm^−1^, 1100 cm^−1^, and 1342 cm^−1^, corresponding to the symmetric, asymmetric, and undefined stretching modes of the C–O–C linkage within the polymer backbone, respectively. Furthermore, peaks at 1466 cm^−1^ and 2881 cm^−1^ are attributed to the vibrational modes of the CH_3_ groups.

As modified, the FT-IR spectrum of F127-COOH retains all the characteristic peaks of the parent F127 polymer. Crucially, the emergence of new absorption bands at 1732 cm^−1^ and 1648 cm^−1^ is observed, assigned to the stretching vibrations of the C=O bonds in the carboxylic acid and ester carboxylate moieties, respectively.

These spectral features provide compelling evidence for the successful conversion of the terminal hydroxyl groups to carboxylic acid groups, thus confirming the desired chemical modification of F127.

**Fig. 8:**
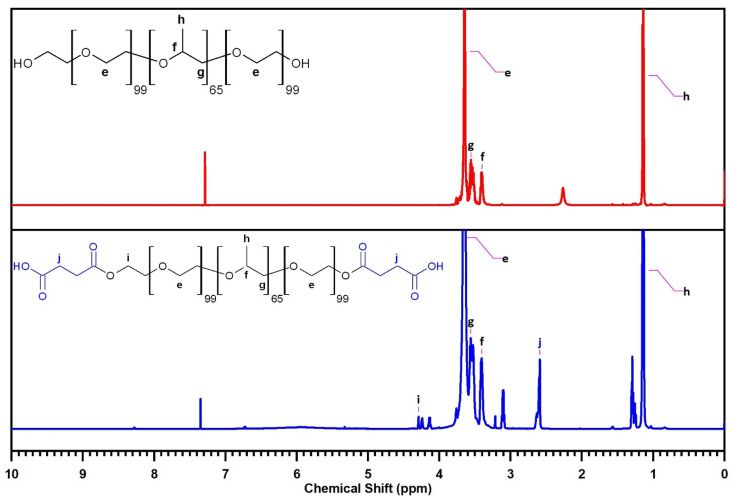
The ^1^H-NMR of F127 (top) and F127-COOH (bottom).

The ^1^H NMR spectrum of F127 (Fig. 8) further corroborates its structural identity through characteristic resonance peaks. Signals at *δ* = 1.13–1.16 ppm, *δ* = 3.40 ppm and *δ* = 3.56 ppm are assigned to the protons (d) of the –C**H**_**3**_, protons (b) of the –C**H**–C**H**_**3**_ groups and protons (c) of the –C**H**_**2**_ groups within the PPO segment, respectively. Resonances in the region of *δ* = 3.55–3.68 ppm are indicative of the protons (a) of the –C**H**_**2**_–C**H**_**2**_– groups in the PEO segment.

The ^1^H NMR spectrum of F127-COOH similarly displays these characteristic peaks associated with the F127 backbone. Crucially, new resonance peaks emerge at *δ* = 2.59–2.63 ppm, attributed to the protons (f) of the –C**H**_**2**_–C**H**_**2**_– groups in the succinic acid moiety, and at *δ* = 4.13–4.29 ppm, characteristic of the protons (e) of the –C**H**_**2**_–COOH groups. The presence of these additional peaks provides compelling spectroscopic evidence for the successful incorporation of the succinic acid moiety into the F127 structure, further confirming the desired chemical modification.

### 3.3. Characterization of F127-GA@ZnO

**Fig. 9:**
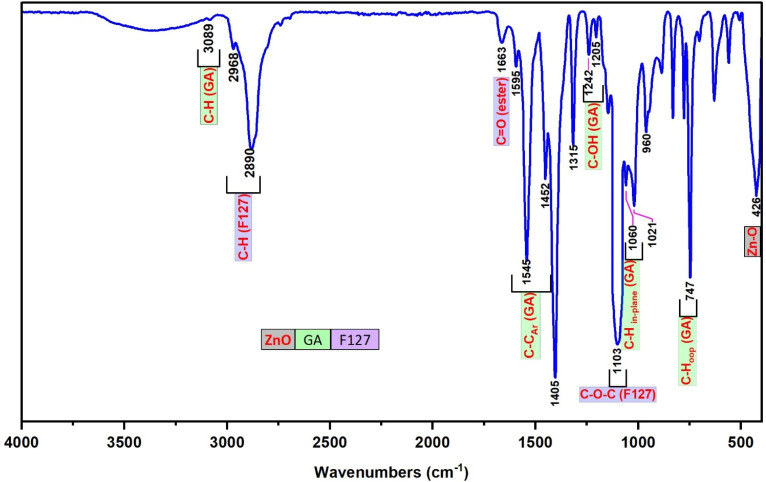
The FT-IR spectra of F127-GA@ZnO.

The FT-IR spectrum of F127-GA@ZnO is presented in Fig. 9. In this spectrum, the peak at 425 cm^−1^ represents the Zn–O bond of the ZnO core. The aromatic ring structure of GA is evidenced by the peaks at 747 cm^−1^ and 777 cm^−1^, attributed to the out-of-plane (oop) bending vibrations of the C–H bonds in the trisubstituted (3,4,5-) aromatic ring of GA, and the peak at 3081 cm^−1^, assigned to the stretching vibration of the aromatic C–H bonds. The peak at 630 cm^−1^ represents the out-of-plane bending vibration, while the peaks in the region of 3256 cm^−1^ – 3307 cm^−1^ are associated with the stretching vibrations of the O–H bonds attached to the aromatic ring. The F127 segment is characterized by peaks at 2882 cm^−1^ and 2969 cm^−1^, corresponding to the stretching vibrations, and peaks at 1452 cm^−1^ and 1316 cm^−1^, representing the bending vibrations of the C–H bonds in the methyl and methylene groups. The peak at 1020 cm^−1^ is attributed to the C–O–C bond, and the peak at 1403 cm^−1^ is a combination band of the C–O and O–H bonds. The carboxylate group in the F127-GA@ZnO structure is characterized by the peak at 1595 cm^−1^, corresponding to the C=O bond in the carboxylate ion, the peak at 1541 cm^−1^ due to the asymmetric stretching vibration, the peak at 703 cm^−1^ characteristic of the scissoring vibration of the –COO– group, and the peak at 1240 cm^−1^ representing the O–C–C bond in the aromatic carboxylate ion structure. Notably, the disappearance of the absorption peaks at 1732 cm^−1^ and 1649 cm^−1^, which represent the C=O bond in the carboxylic group of the F127-COOH molecule, indicates that the carboxylic group of F127-COOH has indeed participated in the reaction to form an ester bond with the hydroxyl group of GA.

**Fig. 10:**
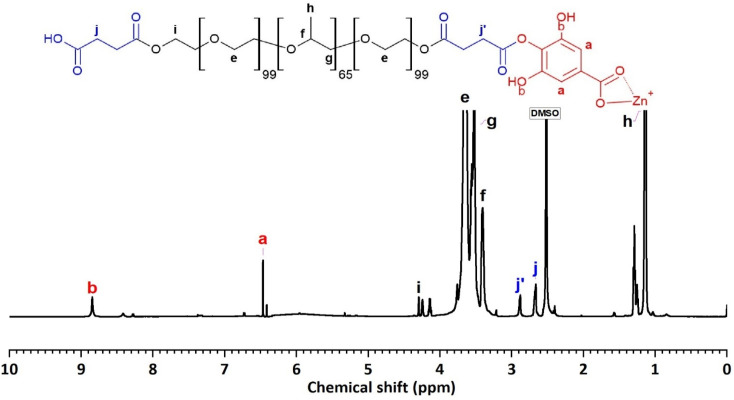
The ^1^H-NMR spectrum of F127-GA@ZnO.

The ^1^H-NMR spectrum (600 MHz, DMSO) of F127-GA@ZnO (Fig. 10) reveals characteristic resonance peaks for the protons of F127-COOH (*δ*_(e)_ = 3.60 ÷ 3.82 ppm; *δ*_(f)_ = 3.41 ppm; *δ*_(g)_ = 3.50 ÷ 3.60; *δ*_(h)_ = 1.11 ÷ 1.17 ppm; *δ*_(i)_ = 4.20 and *δ*_(j)_ = 2.67 ppm), as well as peaks characteristic of GA protons attached to the surface of ZnO NPs (*δ*_(a)_ = 6.47 ppm and *δ*_(b)_ = 8.85 ppm). Furthermore, the appearance of a resonance peak at *δ* = 2.88 ppm can be attributed to proton (j′) when the terminal carboxyl group of F127-COOH forms an ester bond with the hydroxyl group of GA. The aromatic ring structure of the GA molecule exerts a strong electron-withdrawing effect, leading to deshielding of adjacent protons upon ester bond formation with the carboxyl group of F127-COOH. This results in the degeneration of some protons (j) into (j′) and a downfield shift of the (j′) peak. Additionally, the significant decrease in the resonance signal at *δ*_(c)_ = 8.41 ppm suggests that some GA molecules on the nanoparticle surface have participated in the esterification reaction. Analysis of the ^1^H NMR spectrum of GA@ZnO reveals two characteristic proton signals for the hydroxyl groups at chemical shifts (*δ*) of 8.82 ppm and 8.41 ppm, assigned to the protons at the *meta* and *para* positions relative to the carboxyl group, respectively. However, the ^1^H NMR spectrum of F127-GA@ZnO exhibits only one strong signal at *δ* 8.85 ppm and weaker signals at *δ* 8.42 ppm and 8.28 ppm.

This change in the ^1^H NMR spectrum suggests that F127-COOH has been covalently attached to the hydroxyl group of GA at the *para* position. Specifically, the decrease in intensity and slight shift of the hydroxyl proton signal at the *para* position (from 8.41 ppm to 8.42 ppm and 8.28 ppm) indicates a change in the chemical environment around this proton due to the formation of an ester linkage with F127-COOH.

This finding is consistent with the theory of higher reactivity of the *ortho* and *para* positions on the aromatic ring in electrophilic aromatic substitution reactions. Therefore, it can be concluded that F127-COOH preferentially attacks the hydroxyl group at the *para* position of GA on the surface of GA@ZnO.

Thus, the FT-IR and ^1^H-NMR spectral results demonstrate that F127-COOH has formed an ester bond with GA attached to the surface of ZnO nanoparticles, and F127-GA@ZnO has been successfully synthesized.

**Fig. 11:**
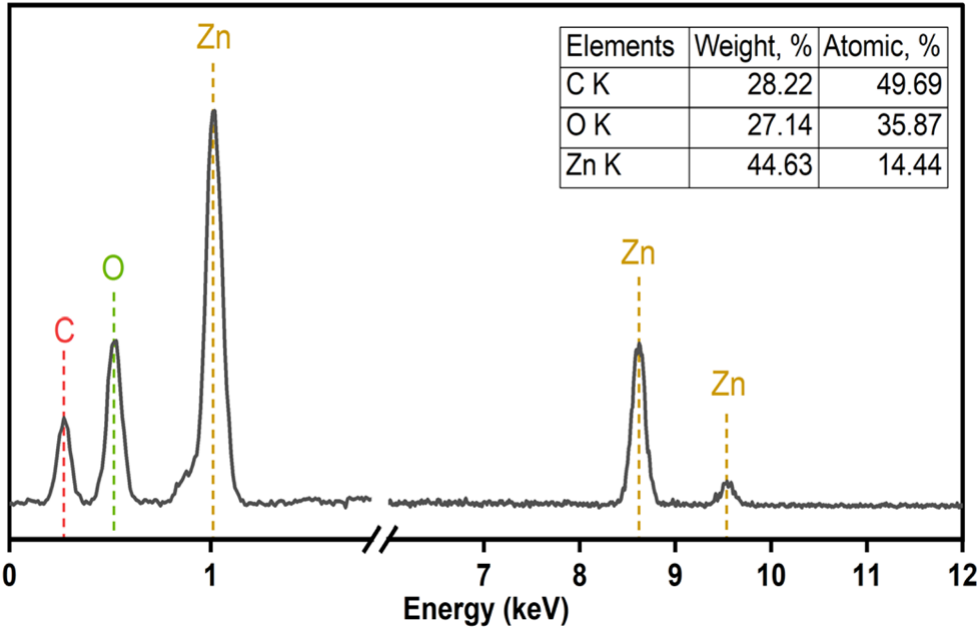
EDS spectra of F127-GA@ZnO.

The elemental composition of F127-GA@ZnO was elucidated through energy-dispersive X-ray spectroscopy, and the corresponding spectrum is presented in Fig. 11. A notable increase in carbon (C) content was observed, reaching 28.22% by mass. This substantial carbon percentage strongly supports the successful incorporation of both the Pluronic F127 polymer and gallic acid onto the surface of the ZnO nanoparticles.

**Fig. 12:**
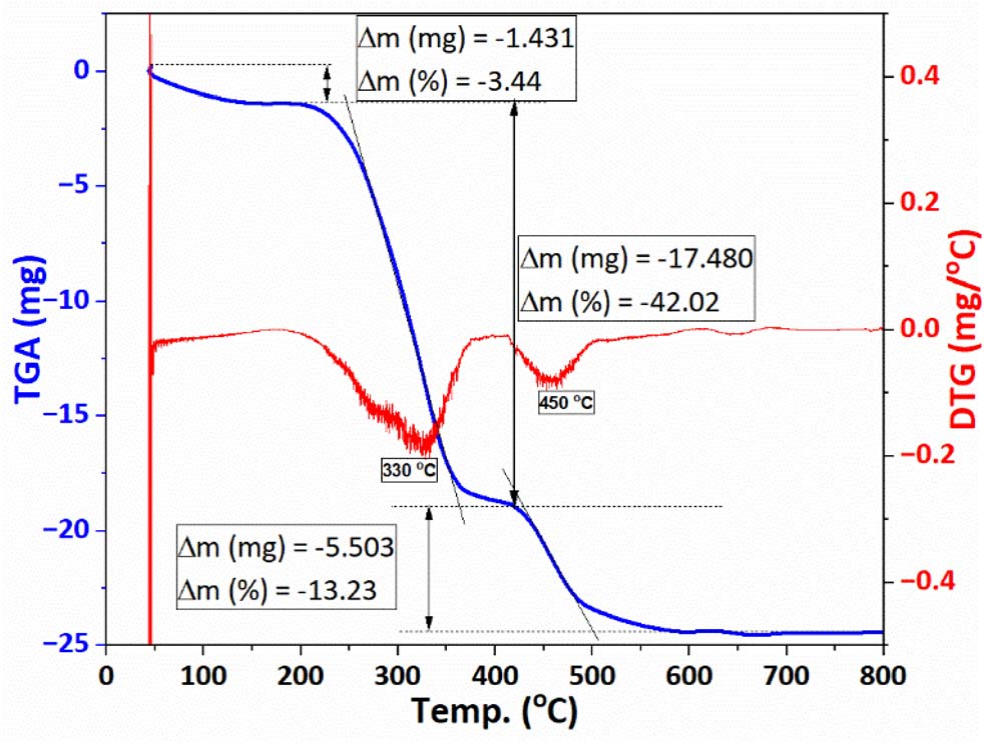
The TGA/DTG diagraph of F127-GA@ZnO.

The TGA/DTG thermogram of F127-GA@ZnO (Fig. 12) exhibits three main weight loss regions. Firstly, from the initial temperature to approximately 150 °C, a weight loss of about 3.44% is observed. This weight loss can be attributed to the evaporation of moisture. The second weight loss region occurs from approximately 200 °C to 400 °C, with a peak observed at 330 °C on the DTG curve. The rate of weight loss is rapid, reaching up to 42.02%. This region of weight loss is responsible for the decomposition of the outermost F127 layer. Finally, we attribute the weight loss region to GA, which spans from approximately 410 °C to nearly 600 °C, and shows a peak on the DTG curve at 450 °C. The weight loss in this region is about 13.23%. This result is consistent with the thermal analysis results of GA@ZnO (Fig. 5).

**Fig. 13:**
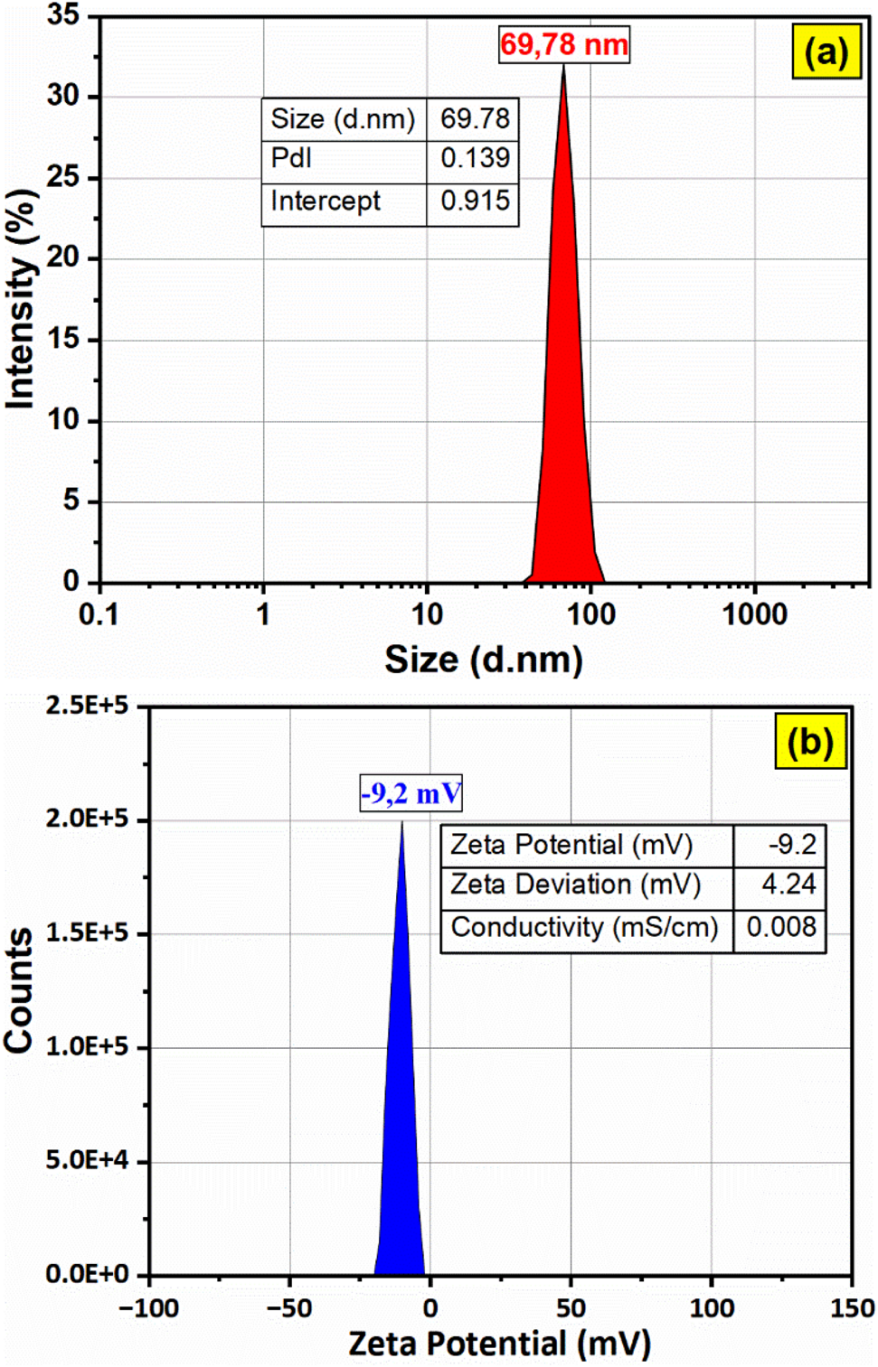
The particle size distribution (a) and zeta potential (b) of F127-GA@ZnO.

The hydrodynamic diameter of the F127-GA@ZnO nanoparticles was assessed *via* dynamic light scattering (DLS), with results depicted in Fig. 13. The DLS analysis revealed a mean hydrodynamic diameter of approximately 69.78 nm for the nanoparticles, accompanied by a narrow peak width and a low polydispersity index (PdI) value of 0.139, indicative of a relatively homogenous size distribution. Furthermore, the high intercept value of 0.915 suggests a strong correlation between the experimental data and the calculated DLS model. Zeta potential measurements indicated a low surface charge of −9.2 mV, contributing to the excellent colloidal stability observed for these nanoparticles. The electrical conductivity was determined to be 0.0084 mS cm^−1^, a considerably low value attributed to the presence of the F127-GA organic layer on the ZnO nanoparticle surface, which acts as an insulating barrier.

**Fig. 14:**
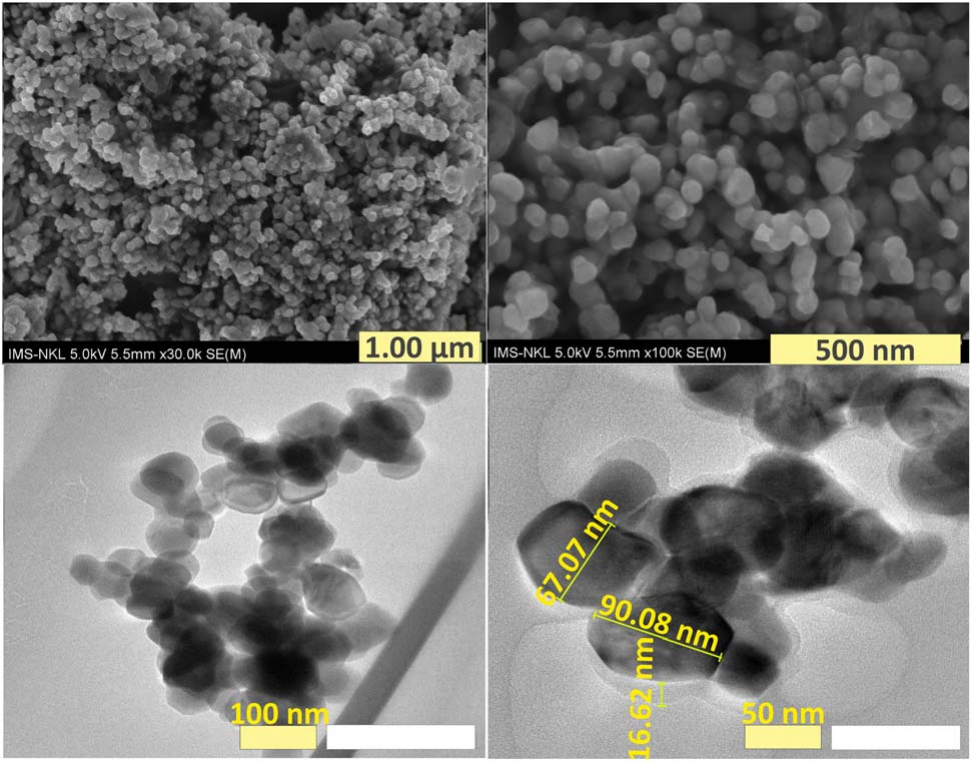
SEM (top) and TEM (bottom) images of F127-GA@ZnO.

The morphology of F127-GA@ZnO nanoparticles was examined using scanning electron microscopy (SEM) and transmission electron microscopy (TEM), and the findings are displayed in Fig. 14. Scanning electron microscopy (SEM) images offer a comprehensive view of the shape and structure of nanoparticles, clearly displaying a protective layer enveloping the nanoparticles. The level of nanoparticle homogeneity stays mostly unaltered. TEM images provide more clarity on the existence of an organic covering surrounding the nanoparticles, which has a low contrast. The nanoparticle sizes were found to be around 67.07 nm and 90.08 nm, which closely matched the size and distribution values obtained using DLS. Moreover, the average thickness of the organic shell enveloping the nanoparticles was found to be approximately 16.62 nm. The combined SEM and TEM findings validate the successful production of nanoparticles consisting of a ZnO NP core surrounded by an F127-GA organic layer.

### 3.4. Evaluation of CPT drug loading capacity of F127-GA@ZnO

**Fig. 15:**
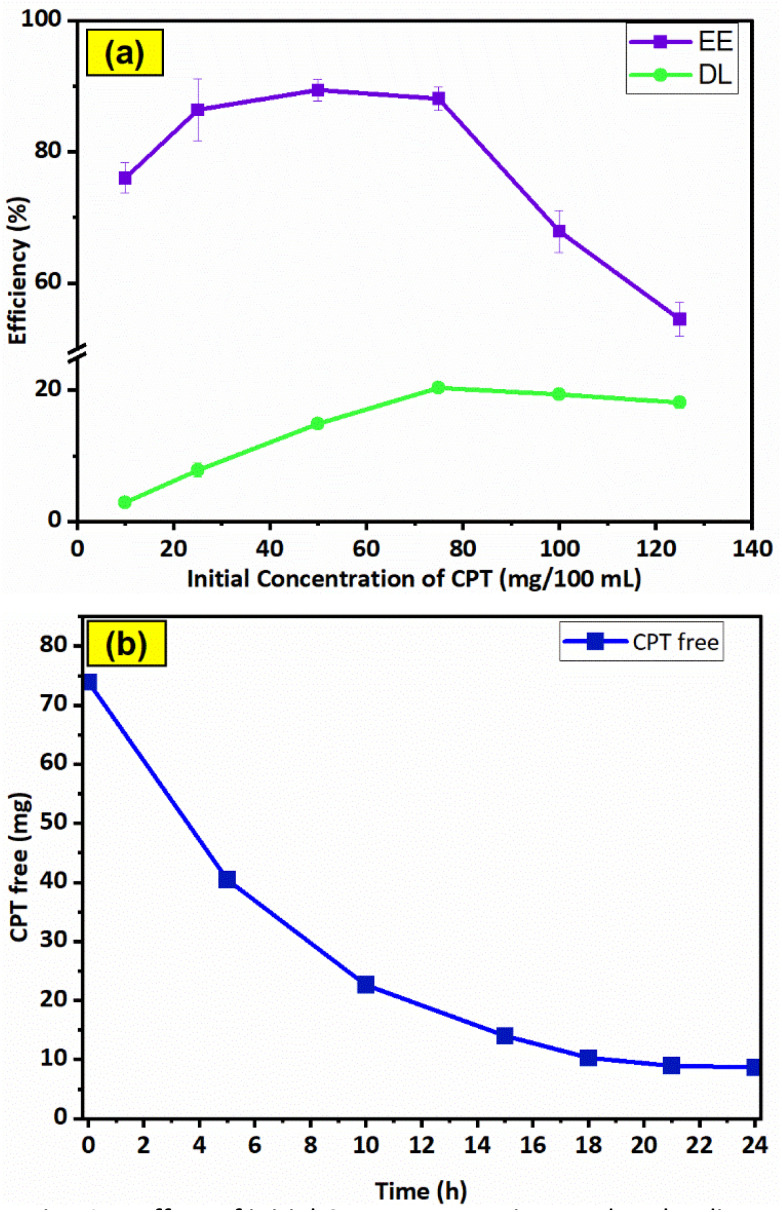
Effect of initial CPT concentration on drug loading efficiency (a) and results of drug loading time investigation (b).

The CPT loading characteristics of F127-GA@ZnO were examined through the dependence of EE and DL on the initial drug content, as shown in Fig. 15a. The data indicates that the initial concentration of CPT significantly influences the encapsulation efficiency (EE) and drug loading (DL). At an initial CPT concentration of 75 mg/100 mL, the highest drug loading (DL) of 20.33 ± 0.8% was achieved, while the encapsulation efficiency (EE) also approached its maximum at 88.13 ± 1.8%, without a significant decrease compared to its highest attainable value.

Therefore, the optimal CPT concentration was determined to be 75 mg/100 mL. To determine the most efficient loading time, the effect of time on the amount of free drug was investigated at the optimal concentration (Fig. 15b). The results show that within the first 10 h, the amount of CPT in the solution decreased rapidly due to the high potential of the loading process, with approximately 30.2% of CPT remaining in the solution. In the subsequent 11 h, the CPT concentration decreased slowly to 13.7% due to the decline in loading potential, while in the last 3 h, the drug amount remained almost constant. Thus, within the 21 hours experimental period, the drug loading reached saturation.

### 3.5. *In vitro* drug release studies

**Fig. 16:**
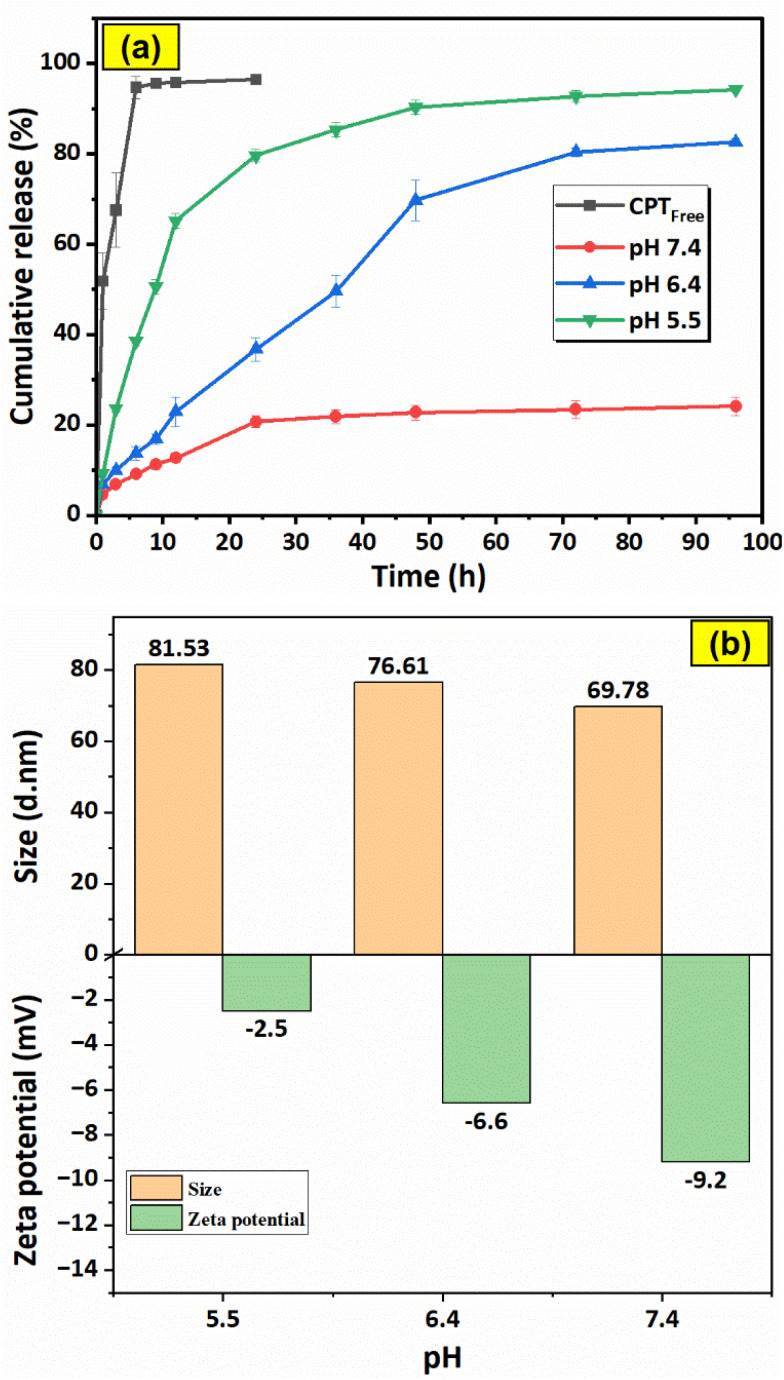
Drug release profiles of free CPT (at pH = 7.4) and F127-GA@ZnO/CPT at various pH (a) and the effect of pH on particle size and zeta potential of F127-GA@ZnO (b). Data are presented as mean ± standard deviation (*n* = 3; *p* < 0.05).

The graphs in Fig. 16a illustrate the drug release characteristics of free CPT (in PBS pH = 7.4) and F127-GA@ZnO/CPT in PBS solutions with varying pH values (pH = 7.4, 6.4, 5.5). The data shows a rapid release of free CPT in physiological buffer, reaching saturation within 6 h with a release efficiency of 94.69%. This suggests a quick equilibrium of drug dialysis and minimal delay in observation. The high release percentage indicates negligible concentration difference between internal and external solutions, ensuring data reliability.

For the F127-GA@ZnO/CPT drug delivery system, a strong dependence on the pH of the environment is observed. At physiological pH, the drug release efficiency is very low and only increases significantly during the first 24 h of the experiment to reach saturation, with the amount of drug released reaching only 20.77 ± 1.24%. The cumulative drug release after 96 h of testing is 24.14 ± 1.98%. When the acidity of the test environment is increased to pH = 6.4, both the release rate and the amount of drug released are significantly improved. The amount of drug released increases continuously during the first 48 h of the experiment, reaching 36.76 ± 2.63% after 24 h and 69.70 ± 4.44% after 48 h. In the subsequent 48 h of testing, the drug release rate is very slow. The cumulative drug release after 96 h of testing reaches 82.54 ± 0.71%.

In an even more acidic environment with pH = 5.5, both the rate and the amount of drug released are further enhanced and can be divided into three phases: the first 12 h of the experiment show a burst release, with the amount of drug released reaching 65.19 ± 1.66%; from 12 h to 48 h, the drug release rate is moderate, with the cumulative drug release after 48 h reaching 90.34 ± 1.53%; and from 48 h to 96 h, the drug release rate is very slow, with a negligible amount of drug released during this phase. After 96 h of incubation, the amount of drug released reaches 94.19 ± 0.48% (Table 1).

**Table 1:** Drug release kinetic parameters of F127-GA@ZnO/CPT

	Korsmeyer–Peppas			Higuchi	
	*K*	*n*	*R* ^2^	*K*_H_	*R* ^2^
pH = 7.4	0.188	0.418	0.972	0.137	0.966
pH = 6.4	0.065	0.615	0.965	0.346	0.989
pH = 5.5	0.137	0.532	0.910	0.422	0.967

Thus, the more acidic the environment, the faster the drug release rate and the larger the amount of drug released. This can be attributed to the attack of H^+^ protons on the bond between the drug and the functional groups (hydroxyl, carboxylic) of the carrier, leading to the massive release of the drug. At even lower pH values, the protons can penetrate the organic shell, reach the surface of the ZnO nanoparticles and break the ZnO-organic layer bond, thus releasing almost the maximum amount of drug. When examining the effect of pH on the particle size and surface potential of the F127-GA@ZnO nanoparticles, interesting changes were observed (Fig. 16b). As the pH decreased, the particle size distribution shifted to larger sizes. This could be due to the H^+^ protons causing swelling of the organic shell and/or changing the morphology of the Pluronic tail on the organic shell of the nanoparticles. It is possible that the Pluronic F127 segments become more extended in acidic environments and facilitate drug access and release into the environment. The zeta potential also tends to shift more positively as pH decreases. This can be reasonably explained by the attack of H^+^ protons, which reduces the number of negative charges on the surface.

The drug release kinetics were also investigated using two mathematical models: Korsmeyer–Peppas and Higuchi. These models are commonly employed to study drug release where the drug–carrier interaction is primarily physical, allowing for consideration of both diffusion and carrier swelling processes. Based on the regression correlation values (*R*^2^), it can be observed that the mathematical models fit the drug release profile of F127-GA@ZnO/CPT reasonably well, particularly the Higuchi model.

**Fig. 17:**
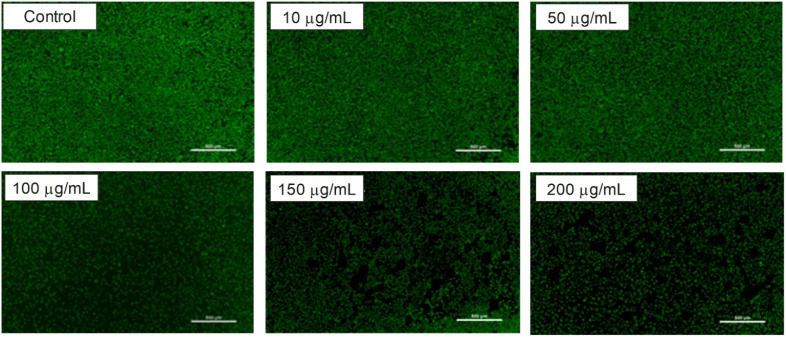
Confocal microscopy images of L929 cells treated with F127-GA@ZnO at different concentrations.

For the Korsmeyer–Peppas model, there is a significant change in the *R*^2^ value as the pH decreases, with lower *R*^2^ values indicating a less suitable fit at lower pH levels. This can be explained by the fact that at low pH, the drug release during this phase involves the breaking of chemical interactions between the drug and the carrier, which deviates from the assumptions of this model. The diffusional exponent (*n*) of this model provides further information about the drug release mechanism. Specifically, when the environment has a lower pH (pH = 6.4 and 5.5), the diffusional exponents are 0.615 and 0.532, respectively (0.45 < *n* < 0.89). According to the interpretation of this model, the drug release process is anomalous (non-Fickian) diffusion, meaning it does not follow traditional Fickian diffusion laws, where the mean squared displacement of particles is not linearly dependent on time.^64^ Several factors can lead to this process, and the most likely explanation in this scenario is the presence of heterogeneity in the interactions between particles. As mentioned earlier, drug release at low pH involves both the release of physically adsorbed drugs (weak bonds) and drugs that have chemical interactions with the carrier (strong bonds).^74^ In contrast, at pH = 7.4, the value of *n* is 0.418, indicating Fickian diffusion as the drug release mechanism. In this case, the released drug is solely the amount physically adsorbed onto the nanocarrier. These results align with the aforementioned explanations (Fig. 17).^64^

### 3.6. Biocompatibility evaluation of F127-GA@ZnO

**Fig. 18:**
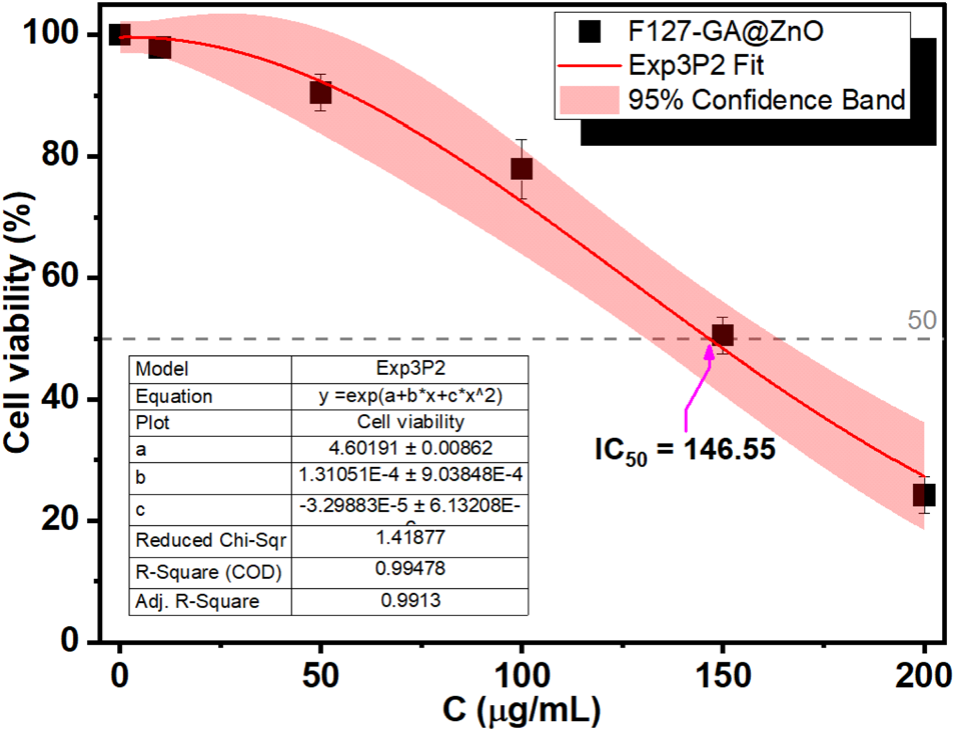
Cytotoxicity of F127-GA@ZnO on L929 cells.

The biocompatibility of F127-GA@ZnO nanomaterials was evaluated by assessing their cytotoxicity towards mouse fibroblast cells (L-929). Fig. 15 presents images of AlamarBlue assays conducted on L-929 cells treated with F127-GA@ZnO at various concentrations for 48 h. Cell viability was determined spectrophotometrically and is presented graphically in Fig. 18. The IC_50_ value, determined by interpolation from the graph, was found to be 146.55 μg mL^−1^.

This high IC_50_ value indicates significant biocompatibility of the drug carrier towards normal cells. In comparison to published studies^75,76^ on the cytotoxicity of ZnO NPs against L-929 cells, F127-GA@ZnO exhibits markedly reduced toxicity. This result confirms that the F127-GA organic coating effectively mitigates the cytotoxic effects of ZnO on healthy cells, as anticipated.

### 3.7. Cancer cells cytotoxicity

**Fig. 19:**
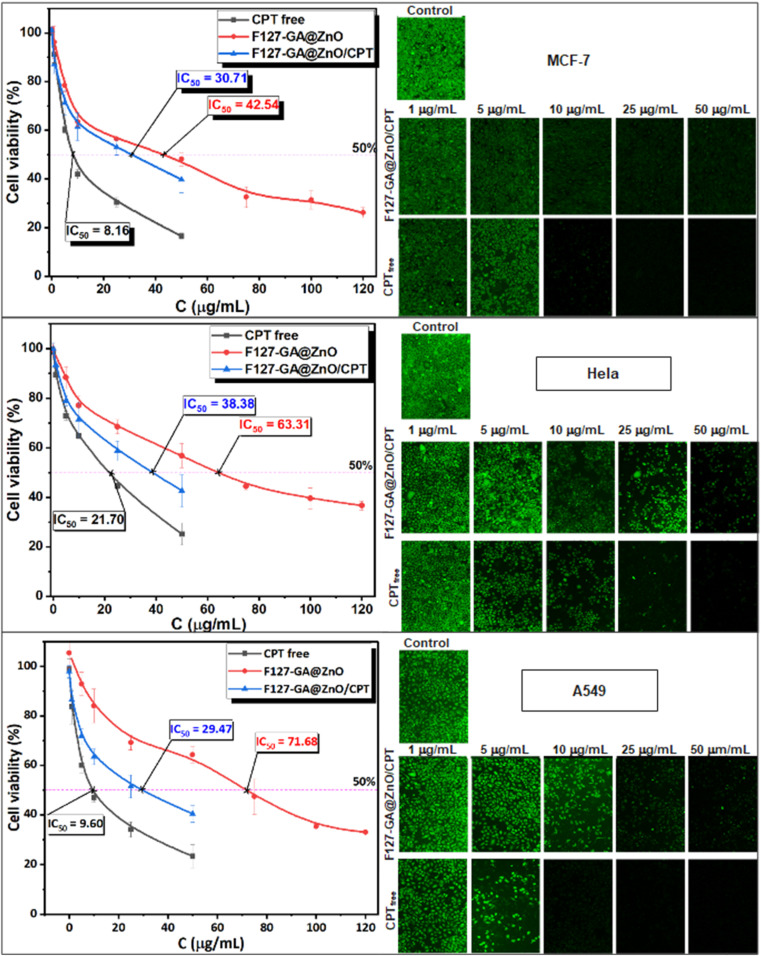
The cell toxicity test findings for free CPT, F127-GA@ZnO, and F127-GA@ZnO/CPT on MCF-7, HeLa, and A549 cancer cells are shown (scale bar = 500 μm). The graph depicts the mean and standard deviation, with a sample size of 3 and a significance level of *p* < 0.05.

The anticancer efficacy of F127-GA@ZnO and F127-GA@ZnO/CPT (drug-loaded) was evaluated against several cancer cell lines, including MCF-7 breast cancer cells, HeLa cervical cancer cells, and A549 lung cancer cells. The cytotoxicity was assessed in comparison to free CPT after 48 h of treatment. Firstly, the experimental results (Fig. 19) indicate that the half-maximal inhibitory concentration (IC_50_) of the free CPT against MCF-7, HeLa, and A549 cancer cells are 8.16 μg mL^−1^, 21.70 μg mL^−1^, and 9.60 μg mL^−1^, respectively.

**Fig. 20:**
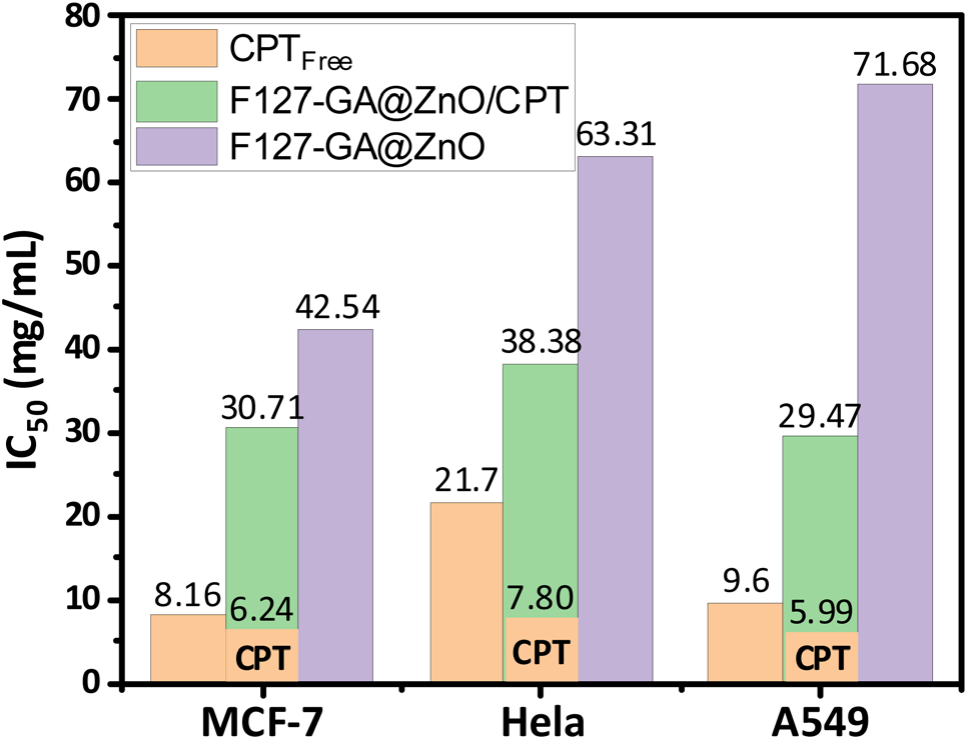
IC_50_ results of CPT free, F127-GA@ZnO/CPT and F127-GA@ZnO against different cancer cell types.

These results are consistent with some previously reported findings.^77–81^ The IC_50_ values of the F127-GA@ZnO carrier were found to be 42.54 μg mL^−1^, 63.31 μg mL^−1^, and 71.68 μg mL^−1^ for the tested cancer cells, respectively. This result indicates that the cytotoxicity towards cancer cells of the ZnO nanoparticles synthesized using guava leaf extract, as in the previous study,^60^ has been improved after surface modification with GA and F127. Meanwhile, the IC_50_ values of the F127-GA@ZnO/CPT drug delivery system for these tested cells were 30.71 μg mL^−1^, 38.38 μg mL^−1^, and 29.47 μg mL^−1^, respectively. It should be noted that this drug delivery system contains only 20.33% CPT by mass. Therefore, the actual IC_50_ values of CPT when loaded onto the F127-GA@ZnO carrier for these cells are calculated to be 6.24 μg mL^−1^, 7.80 μg mL^−1^, and 5.99 μg mL^−1^, respectively, which are 1.30, 2.78, and 1.60 times lower than those of the unencapsulated CPT. This indicates a synergistic effect on the cancer cell killing efficiency of CPT and the F127-GA@ZnO carrier. A comparison of the IC_50_ values of the tested subjects for cytotoxicity against different cancer cells is presented in Fig. 20.

**Fig. 21:**
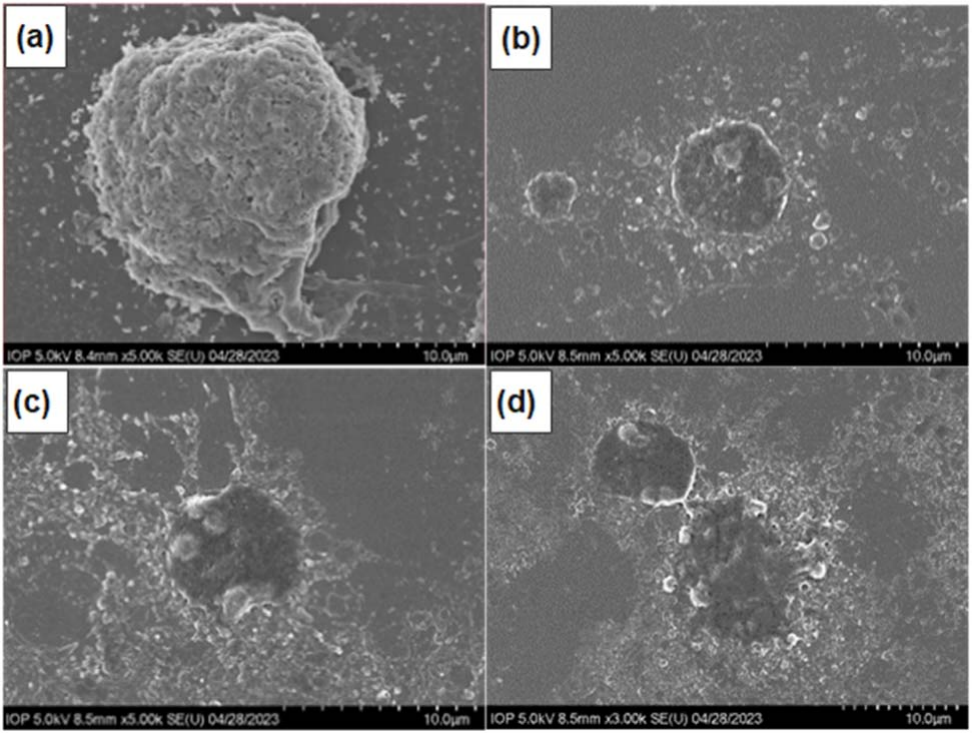
SEM images of control (a) and F127-GA@ZnO/CPT-treated (b–d) A549 cells.

To investigate the mechanism of cell death induced by F127-GA@ZnO/CPT, the morphology of A549 cells was observed using scanning electron microscopy (SEM). SEM images (Fig. 21) revealed significant alterations in cell size and shape, as well as the presence of surrounding bodies. Specifically, A549 cells treated with F127-GA@ZnO/CPT exhibited substantial shrinkage, accompanied by membrane blebbing and the formation of spherical protrusions. Notably, the presence of small cellular fragments, termed apoptotic bodies, was observed in the vicinity of the treated cells.

The formation of apoptotic bodies is a hallmark of apoptosis, distinguishing it from other cell death mechanisms such as necrosis.^82^ During apoptosis, cells undergo a process of shrinkage and fragmentation into membrane-enclosed apoptotic bodies. These apoptotic bodies display “eat-me signals” that attract phagocytic cells, such as macrophages, to clear them, preventing the release of intracellular contents that can trigger inflammation and damage neighboring cells.^83^

## 4. Conclusion

The F127-GA@ZnO, a novel drug delivery material synthesized from guava leaf extract-derived ZnO nanoparticles, was thoroughly characterized, revealing a particle size of 69.78 nm, zeta potential of −9.2 mV, and elemental composition of C (28.22%), O (27.14%), and Zn (44.63%). The material exhibited high CPT drug loading (DL ∼20.33 ± 0.8%, EE ∼88.13 ± 1.8%) and pH-dependent release, with faster release in acidic environments. Cancer cell cytotoxicity assays demonstrated a significant increase in CPT efficacy against cell lines, especially HeLa and A549 cells, which could be attributed to synergistic drug–carrier interactions. While MCF-7 cells showed a smaller increase in cancer cell killing efficacy. We attributed these manifestations to the synergistic anticancer effects of the drug and carrier on the ZnO NPs platform, which has been reported by several research groups, and the acidity of the extracellular environment (pH_e_). The induction of apoptosis in A549 cells by F127-GA@ZnO/CPT was evidenced by distinct morphological changes observed through SEM images. These changes included significant cell shrinkage and the presence of apoptotic bodies, both of which are characteristic features of apoptotic cell death. The findings of this study underscore the immense potential of F127-GA@ZnO as a promising platform for the targeted delivery of CPT in cancer therapeutic applications.

## Data availability

The manuscript includes all data supporting this article.

## Author contributions

Nguyen Ngoc Son: methodology, investigation, data curation, visualization, writing – original draft. Nguyen Thi Huong: conceptualization, formal analysis, project administration, resources, supervision, writing – review & editing. Vu Minh Thanh: funding acquisition, formal analysis, resources, validation.

## Conflicts of interest

The authors declare that they have no known competing financial interests or personal relationships that could have appeared to influence the work reported in this paper.
